# Proton Gradients and Proton-Dependent Transport Processes in the Chloroplast

**DOI:** 10.3389/fpls.2016.00218

**Published:** 2016-02-29

**Authors:** Ricarda Höhner, Ali Aboukila, Hans-Henning Kunz, Kees Venema

**Affiliations:** ^1^Plant Physiology, School of Biological Sciences, Washington State University, PullmanWA, USA; ^2^Departamento de Bioquímica, Biología Celular y Molecular de Plantas, Estacion Experimental del Zaidín, Consejo Superior de Investigaciones CientíficasGranada, Spain

**Keywords:** proton gradient, chloroplast, thylakoid, envelope, cation/H+ exchanger

## Abstract

Proton gradients are fundamental to chloroplast function. Across thylakoid membranes, the light induced -proton gradient is essential for ATP synthesis. As a result of proton pumping into the thylakoid lumen, an alkaline stromal pH develops, which is required for full activation of pH-dependent Calvin Benson cycle enzymes. This implies that a pH gradient between the cytosol (pH 7) and the stroma (pH 8) is established upon illumination. To maintain this pH gradient chloroplasts actively extrude protons. More than 30 years ago it was already established that these proton fluxes are electrically counterbalanced by Mg^2+^, K^+^, or Cl^-^ fluxes, but only recently the first transport systems that regulate the pH gradient were identified. Notably several (Na^+^,K^+^)/H^+^ antiporter systems where identified, that play a role in pH gradient regulation, ion homeostasis, osmoregulation, or coupling of secondary active transport. The established pH gradients are important to drive uptake of essential ions and solutes, but not many transporters involved have been identified to date. In this mini review we summarize the current status in the field and the open questions that need to be addressed in order to understand how pH gradients are maintained, how this is interconnected with other transport processes and what this means for chloroplast function.

## Introduction

Proton (H^+^) gradients across biomembranes represent strong driving forces vital for cellular and cell organellar function. In plant cells, several compartments with different pH exist in parallel. While the apoplast and the vacuole maintain fairly acidic pH levels generally between pH 5 and 7 (Grignon and Sentenac, 1991; Martiniere et al., 2013; Shen et al., 2013). The cytosol has to stay neutral (pH 7.2-7.4) to ensure proper biochemical reactions (Schumacher, 2014). Only mitochondria, the chloroplast stroma and some peroxisomes offer alkaline reaction conditions with pH values equal to or higher than 8 (Shen et al., 2013). The pH gradients have to be actively maintained. In the chloroplast thylakoid membrane or in mitochondria this is achieved by H^+^-transporting electron transfer chains while other organelles depend on ATP fueled H^+^-pumps (ATPases). Although H^+^-pumping activities have been detected in several organellar membranes, the molecular identity of all proteins involved is not known. The established pH-gradients are critical to drive secondary active transport by for instance organellar ion/H^+^ exchange. In recent decades, most research has focused on H^+^-gradient dependent transporters in endomembrane organelles, but knowledge on mitochondria and chloroplasts lags behind (Bassil et al., 2012; Pittman, 2012). Given the central role of mitochondria and chloroplasts for plant energy metabolism, these systems are urgently awaiting more targeted research activity.

This review focuses on the chloroplast, which harbors the pathway of arguably the most important biochemical process for life on earth, photosynthesis. Eukaryotic photosynthesis depends on the H^+^ and ion-gradients established between the sub-organellar chloroplast compartments, the thylakoid lumen and the stroma. We will discuss current knowledge on H^+^-gradients and H^+^-coupled transport across the chloroplast membranes, and how pH-homeostasis in chloroplast compartments is achieved and maintained.

## The H^+^-Gradient Across the Thylakoid Membrane

Light-induced electron transport across the thylakoid membrane gives rise to H^+^-pumping into the thylakoid lumen. The resulting H^+^-electrochemical potential difference (pmf) is used for ATP synthesis by the thylakoid ATP synthase. It is generally assumed that both components of the pmf, that is the pH gradient (ΔpH) and the electric field (ΔΨ), generated by the electron transport chain are equally capable of driving ATP synthesis (Kramer et al., 2003). Although the luminal pH was suggested to drop below pH 5 in some circumstances, recent studies show more modest values of 5.7–7.8 while the stromal pH rises to about 7.8–8.0 (Takizawa et al., 2007). Early reports indicated that the H^+^-accumulation is electrically compensated by K^+^ or Mg^2+^ efflux, or Cl^-^ influx (Enz et al., 1993), but only recently, first transporter genes involved in pH- and osmoregulation in the thylakoid were discovered.

**FIGURE 1:**
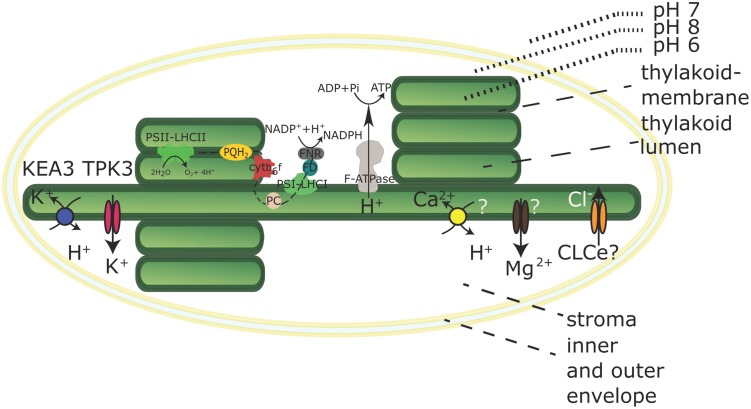
**Chloroplast with its electron transport chain and selected transporters in the thylakoid membrane.** Transport directions are shown for chloroplasts in the light. The K^+^/H^+^ antiporter KEA3 and the Potassium Channel TPK3 are confirmed thylakoid membrane K^+^ transporters. The molecular identity of Ca^2+^ and Mg^2+^ transporters has not been determined and the localization and transport activity of the CLCe channel is uncertain. Both is indicated by question marks. The black dashed line indicates linear electron transport in the thylakoid membrane. Ratios and dimensions of the protein complexes involved in the electron transport chain are schematic and do not reflect physiological conditions.

First, the two-pore potassium channel TPK3 was confirmed by immunodetection to localize to the stroma lamellae (Carraretto et al., 2013; **Figure 1**). TPK3-RNAi-silenced *Arabidopsis* plants revealed stunted growth phenotypes along with anthocyanin accumulation under ambient light conditions (90 μE). TPK3 mutants are compromised in generating ΔpH while increasing ΔΨ by 20% compared to wild-type controls (Carraretto et al., 2013). The channel is *in vitro* activated by Ca^2+^ and acidification and releases K^+^ from the thylakoid membrane to regulate the pmf (Carraretto et al., 2013). Additionally, light-induced Mg^2+^ release into the stroma (Krause, 1977) and luminal Cl^-^ uptake (Enz et al., 1993) was demonstrated. However, the identity of the Mg^2+^ transporter at the thylakoid membrane remains hypothetical (**Figure 1**). T-DNA mutants of the thylakoid membrane channel AtCLCe reveal only subtle effects on photosynthesis (Marmagne et al., 2007), and a role in charge equilibration or Cl^-^ import into the lumen still remains hypothetical as indicated in **Figure 1**. Thus, K^+^ appears to be the fundamental coupling ion.

Recently, an electro-neutral K^+^ efflux antiporter (KEA3) from the Cation Proton Antiporter (CPA2) family was identified in the thylakoid membrane, predominantly in stroma lamellae (**Figure 1**), of *Arabidopsis* (Armbruster et al., 2014; Kunz et al., 2014). Independent *kea3* T-DNA insertion mutants reveal an increased ΔpH component of pmf partitioning with a 20% diminished ΔΨ, suggesting a role in pmf regulation via K^+^/H^+^ exchange across the thylakoid membrane (Kunz et al., 2014). An increase in conductivity to counter ions, for instance by opening of the TPK3 channel, will promote the development of the ΔpH component, but short-circuit the membrane potential, while the activity of KEA3 would only dissipate the ΔpH component, leaving the ΔΨ intact. Armbruster et al. (2014) showed a crucial role for KEA3 in altering the dynamics of the non-photochemical quenching (NPQ) component qE under fluctuating light conditions. qE dissipates excessive excitation energy into heat and is triggered by low lumen pH (Muller et al., 2001). KEA3 was shown to increase photosystem II (PSII) quantum yield [φ(II)] during the transition from high light to low light by accelerating the downregulation of qE via H^+^-efflux from the lumen (Armbruster et al., 2014). At the same time, reduced luminal acidification in plants with impaired TPK3 function causes increased sensitivity to ambient light intensities, as the mechanisms to dissipate excess light energy cannot be activated (Carraretto et al., 2013). A similar observation was made for cyanobacterial TPK3 counterpart SynK (Checchetto et al., 2012). In a SynK-deficient mutant, the decreased ΔpH component also leads to the inability to trigger NPQ. Thus, it appears that the coordinated activation and deactivation of TPK3 and KEA3 enables the interconversion of ΔΨ and ΔpH in response to environmental stimuli, in order to optimize photosynthesis (Kramer et al., 2003; Armbruster et al., 2014).

Genes with high sequence similarity to KEA3 exist in green algae, moss and higher plants, with one gene copy appearing in both *Chlamydomonas* and rice, and two gene copies in *Physcomitrella* (Chanroj et al., 2012). The cyanobacterium *Synechocystis* sp. expresses a CPA2 gene NhaS3, with some sequence similarity to CHX members and KEA3 from *Arabidopsis* (Chanroj et al., 2012). NhaS3, as a potential Na^+^ (K^+^)/H^+^ antiporter was shown to be located in the thylakoid membrane and to enhance K^+^ uptake when expressed in *Escherichia coli.* The authors therefore concluded that NhaS3 is involved in thylakoid lumen ion-homeostasis (Tsunekawa et al., 2009).

It was reported that the thylakoid ΔpH also energizes Ca^2+^ import across the thylakoid membrane (Ettinger et al., 1999). Ettinger et al. (1999) suggested that Ca^2+^ import is driven by a hypothetical Ca^2+^/H^+^ antiporter (**Figure 1**), the gene of which remains elusive.

In summary, the ΔpH component of the pmf contributes to the ATP production, import of proteins, and the uptake of essential ions such as Ca^2+^. Further, the partitioning of pmf in ΔpH and ΔΨ is regulated via transport activities of KEA3 and TPK3, which has fundamental impact on photosynthetic efficiency and other pH-dependent processes. Finally, thylakoid H^+^-transport establishes the physiological pH in lumen and stroma, thereby ensuring the activity of the light-dependent photosynthetic reactions and the Calvin Benson cycle enzymes.

## The H^+^-Gradient Across the Chloroplast Envelope Membrane

**FIGURE 2:**
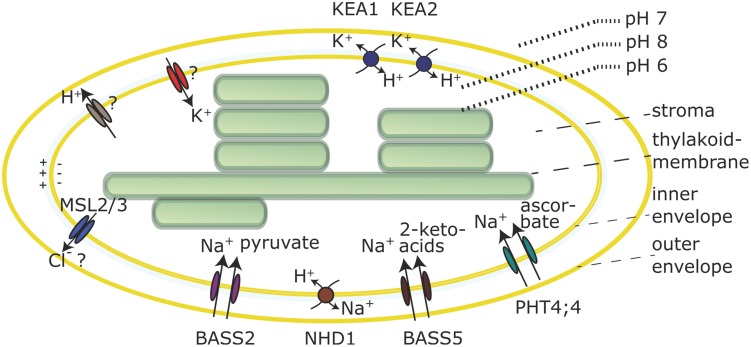
**Selected transporters in the chloroplast envelope membrane.** Transporter activities are shown for chloroplasts in the light. The nature of the H^+^ efflux mechanism and the molecular identity of K^+^ channels is still unknown and the Cl^-^ substrate specificity of the MSL2/3 channels is not confirmed. Both is indicated by question marks.

In the dark the cytoplasmic and stromal pH are close to 7, but upon illumination the stroma becomes alkaline, as a consequence of H^+^-pumping across the thylakoid membrane. An alkaline stroma pH is a prerequisite for the full activation of pH-dependent Calvin Benson cycle enzymes (Heldt et al., 1973; Werdan et al., 1975). This implies that a pH gradient between the cytosol (pH 7) and the stroma (pH 8) is established upon illumination. It is assumed, that in order to maintain this pH gradient, an active H^+^-export mechanism exists in the envelope membrane to compensate for passive H^+^-diffusion from cytosol to stroma. Evidence for an active H^+^-extrusion is based on the observation that illumination of isolated chloroplasts induces a small transitory acidification in slightly buffered solutions (Heber and Krause, 1971; Heldt et al., 1973; Heber and Heldt, 1981; Demmig and Gimmler, 1983). Experiments on equilibration of lipophilic cations and tracer studies in isolated chloroplasts indicate that a membrane potential of around -100 mV (negative inside, **Figure 2**) exists across the envelope membrane due to a Gibbs Donan equilibrium, in low salt solutions. At higher KCl concentrations, similar to the situation inside the cell, this membrane potential becomes very small (Demmig and Gimmler, 1983). In illuminated chloroplasts the membrane potential increases by about 10 mV, concomitant with light induced H^+^-extrusion and coupled K^+^ uptake (Demmig and Gimmler, 1983; Wu et al., 1991). The K^+^/H^+^ exchange is not abolished by the K^+^ ionophore valinomycin, indicating that K^+^ and H^+^-fluxes are electrically and thus indirectly coupled (Demmig and Gimmler, 1983; Wu and Berkowitz, 1992; Wang et al., 1993). Several mechanisms have been proposed to explain the H^+^-efflux, but the molecular identities of the transporters involved are still elusive.

H^+^-extrusion was suggested to depend on a K^+^-stimulated vanadate sensitive envelope P-type H^+^-ATPase (Scherer et al., 1986; Berkowitz and Peters, 1993; Mi et al., 1994; Shingles and McCarty, 1994) but the *Arabidopsis* genome does not encode for chloroplast predicted P-type H^+^-pumps (Axelsen and Palmgren, 2001). Other reports found that the envelope ATPase activity is sensitive to oligomycin, which is not a P-type ATPase inhibitor but a F*_o_* subunit inhibitor of the F_1_F*_o_* ATP Synthase. However, no ATPases were identified in chloroplast proteomics studies of envelope membranes. In conclusion the existence of an envelope H^+^-ATPase is hypothetical. Alternatively, an electron-transfer chain may be responsible for H^+^-extrusion. Although proteins that could fulfill such functions are found in the envelope membrane, their function in H^+^-extrusion has never been demonstrated (Jäger-Vottero et al., 1997; Murata and Takahashi, 1999; Kovacs-Bogdan et al., 2010). In algae, the plastome-encoded, putative heme-binding, inner envelope protein YCF10 from *Chlamydomonas* is essential for enhancing carbon fixation and inorganic carbon uptake (Rolland et al., 1997). The YCF10 protein was suggested to be involved in plastid pH regulation and to be part of a redox chain in the envelope membrane (Jäger-Vottero et al., 1997; Rolland et al., 1997). In *ycf10* deficient mutants of *Chlamydomonas* the CO_2_ or HCO_3_^-^ uptake was compromised and cells failed to grow photoautotrophically (Rolland et al., 1997). It was hypothesized that YCF10 is crucial for acidification of the intermembrane space by H^+^-translocation to allow for the conversion of HCO_3_^-^ to CO_2_, which in turn could diffuse into the chloroplast (Rolland et al., 1997). A similar observation was made for the cyanobacterial homolog PcxA (CotA) that was shown to be involved in light-induced Na^+^-dependent H^+^-extrusion. The *cotA* insertion mutant MA29 of the cyanobacterium *Synechocystis* sp. shows no H^+^-export leading to a diminished CO_2_ uptake (Katoh et al., 1996).

Lastly, it was speculated that an alkaline stromal pH could be maintained by direct H^+^-export from the thylakoid lumen to the cytosol by some unknown temporary or spatial connection (Heber and Heldt, 1981). In summary, the mechanism by which H^+^ move across the envelope membrane and if this involves an ATP-dependent H^+^-pump or electron transport coupled H^+^-export into the intermembrane space or some other mechanism remains unknown (**Figure 2**).

## H^+^-dependent transport mechanisms across the chloroplast envelope membrane

Although the mechanism by which the envelope pH-gradient is generated and maintained during the day remains unclear, it represents the driving force for secondary active H^+^-coupled transport processes across the inner envelope membrane. Thus, it is worthwhile to focus at the characterized and putative H^+^-dependent transport mechanisms and their transport direction across the inner envelope membrane.

Electrogenic coupling of K^+^ uptake to H^+^-extrusion in chloroplasts has been demonstrated many times, and the presence of a K^+^ channel in the envelope membrane was hypothesized (**Figure 2**) based on biochemical studies with membrane vesicles (Wang et al., 1993) as well as patch clamp studies (Heibert et al., 1995). The chloroplast inner envelope is also reported to contain non-selective cation channel activities (Pottosin et al., 2005). However, up to today the molecular identity of these channels is unknown. Instead several, most likely electroneutral, K^+^/H^+^ or Na^+^/H^+^ antiporters were identified. The envelope pH-gradient dictates antiport activity via such antiporters toward K^+^-efflux, which can be an important feature for plastid osmoregulation (Shabala and Pottosin, 2010). Light induced shrinkage of chloroplasts was observed more than 40 years ago (Nobel et al., 1969). K^+^/H^+^ antiporters could be involved in this response, together with mechanosensitive channels MSL2 and MSL3 for which Cl^-^ efflux activity was hypothesized (Veley et al., 2012; **Figure 2**).

Two members of the K^+^/H^+^ antiporter family KEA, KEA1 and KEA2 were confirmed to reside in the envelope membrane of *Arabidopsis* (Kunz et al., 2014). A short version of the KEA2 protein comprising the antiport domain and the regulatory C-terminal KTN domain, but not the long N-terminal domain of unknown function (Chanroj et al., 2012) was purified and reconstituted in liposomes containing the pH indicator pyranine to demonstrate ΔpH-dependent K^+^/H^+^ antiport (Aranda-Sicilia et al., 2012). KEA1 and KEA2 represent very close homologs, most likely due to a gene-duplication event and thus are expected to facilitate the same transport function. Moreover, single T-DNA insertion *kea1* or *kea2* mutants do not show visible phenotypes indicating functional redundancy in *Arabidopsis* (Kunz et al., 2014). *kea1kea2* double mutant plants revealed stunted growth, pale green leaves and low photosynthetic efficiency (Kunz et al., 2014). Chloroplasts from double mutant plants have a swollen appearance, suggesting they suffer from excessive K^+^ accumulation. Interestingly, this phenotype depends on the leaf developmental stage, i.e., young leaves were found to be most strongly compromised (Kunz et al., 2014). Additionally, since the pmf across the thylakoid membrane depends not only on light but also on the stromal pH (Hauser et al., 1995), the lack of K^+^/H^+^-exchange across the envelope membrane in *kea1kea2* mutants probably causes downstream effects that trigger changes in pmf partitioning with lower ΔpH and in turn lower qE (Kunz et al., 2014). This further emphasizes the linkage between plastid ion and pH homeostasis, and their importance for efficient photosynthesis.

The rice chloroplast envelope KEA homolog is encoded by a single locus named *AM1*, (*albino midrib mutant1)*. The *am*1 mutant was isolated in an EMS screen and displays green- and white leaf variegations (Sheng et al., 2014). Surprisingly, no photosynthetic alterations were found in *am1* mutants. However, mutants did reveal altered chloroplast ultrastructure along with increased drought tolerance (Sheng et al., 2014). The authors attribute *AM1* function in rice to chloroplast development and drought stress (Sheng et al., 2014). Interestingly, the growth and photosynthesis defects in *Arabidopsis kea1kea2* double mutant plants could be rescued partially in high NaCl concentrations. Salt or drought stress are known to increase cytoplasmic and vacuolar osmotic values due to accumulation of salt or synthesis of osmolytes. Possibly, this counteracts the increased osmotic values of the chloroplast stroma in absence of these KEA antiporters (Kunz et al., 2014).

In addition to the KEA K^+^/H^+^ antiporters, another cation exchanger was identified in the plastid envelope membrane of *Arabidopsis*, the Na^+^/H^+^ exchanger NHD1 (Furumoto et al., 2011; Muller et al., 2014; **Figure 2**). NHD1 is an NHAD type carrier that is also found in algae, mosses and other plant species (Barrero-Gil et al., 2007; Cosentino et al., 2010). AtNHD1 and homologs from *Mesembryanthemum crystallinum* and *Physcomitrella* were shown to be localized in the envelope membrane (Barrero-Gil et al., 2007; Cosentino et al., 2010; Muller et al., 2014). Investigation of the *Arabidopsis* T-DNA insertion mutant *nhd1-1* showed compromised φ(II) and higher NPQ in response to a NaCl shock treatment (Muller et al., 2014). Proteomics studies on isolated *Arabidopsis* thylakoid membrane fractions also detected NHD1 in the stroma lamellae (Tomizioli et al., 2014). Therefore, a dual localization of the protein within the chloroplast cannot be excluded at the moment.

H^+^-gradient driven chloroplast Na^+^-efflux by NHD1 could prevent Na^+^-accumulation in the organelle during salt stress. Alternatively, this activity could be coupled to the action of Na^+^-dependent transporters in the envelope membrane. As such, AtNHD1 was suggested to operate as a two-translocator system together with the sodium:pyruvate cotransporter BASS2 (Furumoto et al., 2011; **Figure 2**). Several more Na^+^-dependent carriers in the envelope membrane have been described that facilitate ascorbate import (PHT4;4; Miyaji et al., 2015) or 2-keto acids transport (BASS5; Gigolashvili et al., 2009; Sawada et al., 2009; **Figure 2**). For PHT4;4 and BASS2 experimental evidence from *in vitro* transport studies have revealed the Na^+^-dependency. Furthermore, transporters of the PHT2 and PHT4 family, mediate H^+^ and/or Na^+^-dependent transport of phosphate into chloroplasts and are localized in the envelope membrane (Ferro et al., 2002; Versaw and Harrison, 2002; Roth et al., 2004; Guo et al., 2008).

In diatoms that evolved by secondary endosymbiosis, plastids possess four envelope membranes. To supply the plastid gene expression machinery with nucleotides and provide energy-rich components during the absence of photosynthesis, diatoms utilize nucleotide carriers that efficiently shuttle the compounds from the cytosol, where they are synthesized, into the stroma across the four membranes. Unlike the plastid nucleotide transporters (NTTs) from plant and algae, NTT1 from the diatoms *Thalassiosira pseudonana* and *Phaeodactylum tricornutum* is suggested to enter the innermost plastid envelope and function as H^+^-dependent symporter, which implies a vital function for an envelope pH-gradient in the diatoms (Ast et al., 2009).

Biochemical studies on intact isolated chloroplasts have suggested further H^+^-dependent transport processes across the envelope membrane. Measurements on inner envelope membrane vesicles revealed that Ca^2+^-uptake was stimulated by an electrochemical H^+^-gradient across the membrane. Further, Ca^2+^-movement across the membrane could be shown in the presence of a K^+^-diffusion potential gradient (Roh et al., 1998). Two potential candidates were suggested as membrane potential driven Ca^2+^-transporters in the chloroplast envelope. However, for neither of them the Ca^2+^ transport activity or the substrate specificity could be proven and thus the genetic loci encoding for an H^+^/Ca^2+^ uptake mechanism remains unknown. In summary, there is little doubt that Ca^2+^ is crucial for photosynthesis *per se* and therefore fine-tuned delivery systems into stroma and thylakoid lumen have to exist, reviewed in (Hochmal et al., 2015). Because of the significance of Ca^2+^ transporters for chloroplast function this issue needs more detailed research in the future.

It is of note that further transporters functionally important for chloroplast ion homeostasis exist in the envelope membrane. However, since these transporters are not driven by ΔpH, they are not listed here, but are reviewed comprehensively in a recent article (Finazzi et al., 2015).

## Author Contributions

All authors listed, have made substantial, direct and intellectual contribution to the work, and approved it for publication.

## Conflict of Interest Statement

The authors declare that the research was conducted in the absence of any commercial or financial relationships that could be construed as a potential conflict of interest.
